# Description of the healthy eating indices-based diet quality in Turkish adults: a cross-sectional study

**DOI:** 10.1186/s12199-017-0613-z

**Published:** 2017-03-20

**Authors:** Eda Koksal, Merve Seyda Karacil Ermumcu, Hande Mortas

**Affiliations:** Faculty of Health Sciences, Department of Nutrition and Dietetics, Gazi University, Ankara, Turkey

**Keywords:** Healthy Eating Index-2005, Healthy Eating Index-2010, Diet quality, Health, Diet

## Abstract

**Objectives:**

This study aims to describe the dietary status of Turkish adults using two different versions of the Healthy Eating Index (HEI).

**Methods:**

In this cross sectional study, 494 healthy participants (311 females) with randomly selected and living in Ankara were included between September 2013 and March 2014. A questionnaire was completed and anthropometric measurements (weight and height) were performed. The 24-h dietary recall of individuals was collected. Diet quality was measured through HEI-2005 and HEI-2010 scores.

**Results:**

The mean age, body mass index (BMI), HEI-2005 and HEI-2010 scores of individuals were 32.9 ± 10.8 years; 25.0 ± 4.8 kg/m^2^; 56.1 ± 13.9 and 41.5 ± 13.7 points, respectively. Significant differences were found between mean HEI-2005 and HEI-2010 scores (*p* < 0.05). The individual’s whose diet quality needs to be improved according to mean HEI-2005 score, had poorer diet based on mean HEI-2010 scores. The highest mean HEI-2005 and HEI-2010 scores were stated in female, in subjects had low education levels, aged 51 years or older and in overweight groups (*p* <0.05). Both versions of healthy eating indices were correlated positively with BMI and age

**Conclusion:**

Diet qualities of the individuals are associated with age, gender, education and BMI. Although the components and scores in HEI-2010 version were changed from the version of HEI-2005, the changes may encourage healthy choices of some food group. HEI-2010 gives more attention to food quality than HEI-2005. Thus, in the present study it was concluded that HEI-2010 provided more precise results about diet quality.

## Introduction

Many health problems including dyslipidemia, obesity and metabolic syndrome are found to be related to poor and unbalanced diet in recent years [1–4]. The health status of individuals is influenced by the total dietary intake in addition to one nutrient [5, 6]. There have been many studies about single nutrients [7, 8], various foods [9–11] and food groups [12, 13] to achieve etiology of diseases related to nutrition. Similarly, many researchers have investigated diet quality and health interactions [6, 14–17] while in Turkey, there are a few studies investigated the diet quality, especially performed using the diet quality indices [18–21]. The indices used to evaluate the diet quality were developed to measure the degree of compliance with a standard which is considered a healthy diet [22]. A diet quality index measures most important dietary components and scores depending on these components. The assessment of adequacy, diversity and proportionality of diet is carried with diet quality indices [23, 24].

Today, individuals can consider their own diet to have high quality due to low levels of diet quality information with some objective evaluation methods [25]. Therefore, it is very important that correct assessment of individuals’ diet quality. Healthy eating indices are indicated as important tools to assess compliance diet quality with dietary guidelines [22]. Various healthy eating indices are developed in order to reveal the interaction between diet quality and health [26]. Healthy Eating Index (HEI) is among the leading indices assessing the diet quality to maintain health and improve well-being. It is updated at every five years [22, 24, 27]. HEI score was found to be negatively associated with diseases such as abdominal obesity, hypertension, cancer in many studies [22, 23, 28].

In our present knowledge this study is the first one conducted in Turkey to evaluate diet quality using HEI-2010. In this study, in addition to describe diet quality of individuals using two different versions of HEI, it was aimed to investigate influence of baseline factors such as body weight, age, gender and education level which may affect the diet quality.

## Materials and methods

### Study design and sample

In this cross sectional study, 494 healthy participants (311 females) randomly selected were included between September 2013 and March 2014 (the response rate of the study was 76%). The study was carried out in Ankara, the capital city of Turkey. Each participant signed a voluntary participation form and filled in the questionnaires in accordance with the declaration of Helsinki (World Medical Association). Questionnaire including the demographic and general characteristics (age (year), education (illiterate, no schooling-literate, basic/primary school, secondary school, high school and university which are classified according to duration (year)) and body mass index (BMI) was administered to individuals by face-to-face interviews. All anthropometric measurements were taken by two trained dieticians. Height was measured to the nearest 0.1 cm, and weight to nearest 0.5 kg in light clothing and without shoes. BMI was calculated as body weight (kg)/[height (m)]^2^. After BMI was calculated, the results were classified as underweight, normal and overweight according to World Health Organization’s classification [29]. The threshold of education level was set as 8 years, the compulsory education level in Turkey.

### Assessment of diet quality

The 24-h dietary recall was obtained to determine the diet quality of the participants. Nutrition Information Systems (Beslenme Bilgi Sistemi-BeBiS) which is a food software program in compliance with Turkish food was used for assessment nutrients, food and food groups. Further HEI-2010 and HEI-2005 were used for evaluation of the diet quality. These indices consist of twelve components namely nine adequacy and three proportionality components, takes into account the consumption of both healthy and non-healthy foods [22].

### Calculation of the HEI-2005 and HEI-2010 scores

The intakes of foods and nutrients are represented on a density basis, as amounts per 1,000 cal for each index. According to HEI-2005, whole and total fruits, total vegetables, dark green and orange vegetables and legumes, total and whole grains, milk, meat and legumes, oils were scored from 0 to 20 for consumption amounts per 1000 kcal. Saturated fats, sodium and calories from solid fat, alcoholic beverages and added sugars (SoFAAS) were awarded for lower intakes. These components were scored in reverse. With all scores collected were reached the total HEI-2005 score. The collected HEI score can potentially range from a minimum of zero to a maximum of 100. Total HEI-2010 score was calculated similar to HEI-2005. But there were some updated or added foods in HEI-2010. “Dark green and orange vegetables and legumes” component was changed as “green and beans”. “Milk” component was changed as “dairy”. “Meat and beans” component was changed as “total protein foods”. “SoFAAS” component was changed as “empty calories”. Total grain was eliminated. Also, “seafood and plant protein” and “fatty acids and refined grains” components were added. There were made no change in total scoring and was defined as the maximum score of 100 [30]. Possible scores range from 0 to 100, with 100 points referring to perfect diet quality and lower results indicating larger deviations from the recommended intakes. Diet quality indices were categorized into three stages. A total score of ≤50 was described as “poor diet quality”, scores of 51–80 considered “needs improvement” and scores of >80 indicated “good” [18, 30]. Comparison of components and scoring standards in the HEI-2005 and HEI-2010 was shown Table 4 [31].

### Statistical analysis

Assessment of the study data was carried out using Statistical Package for the Social Sciences 16.0 (SPSS). Independent groups were analyzed for mean differences and significance of data providing parametric conditions (ANOVA, *t* test and Mann–Whitney *U* test). The Chi-square test was used to compare the proportions in different groups. The correlation coefficients and significance were calculated using Pearson test. Moreover, the inter-rater agreement between the two indices in determining the diet quality as poor and needed improvement was investigated using the Kappa test. A *p*-value of less than 0.05 was considered to show a statically result.

## Results

### Socio-demographic and nutritional characteristics

Socio-demographic and nutritional characteristics of the participants were presented in Table 1. Mean age and BMI of individuals were 32.9 ± 10.8 years; 25.0 ± 4.8 kg/m^2^, respectively (data not shown in the tables). The mean age of females and males were 33.5 ± 10.2 and 31.9 ± 11.7 years, respectively (Table 1). Females (25.1 ± 5.2) had higher mean BMI than males (24.9 ± 4.0) but difference between them was not significant (*t = −*0.553; *p* = 0.580). In total, 17.0% of females and 24.0% of males were university graduates. Also, the proportion of the individuals who were secondary school graduates (compulsory education level in Turkey) was higher in males than females (13.1 and 9.3%, respectively). In general, significant differences were found between the males and females without the mean daily intakes of vitamin A (μg), vitamin E (mg) and vitamin C (mg). The daily intake of energy and total protein was 1906.5 kcal and 69.1 g in males and 1571.4 kcal and 52.3 g in females.

**Table 1:** Socio-demographic characteristics, daily energy and nutrient intakes of the participants

	Males (*n* = 183)	Females (*n* = 311)
Age (years), mean (SD)	31.9 ± 11.7	33.5 ± 10.2
	*t = −1.540; p = 0.124*	
BMI (kg/m^2^), mean (SD)	24.9 ± 4.0	25.1 ± 5.2
	*t = −0.553; p = 0.580*	
Educational status, *n* (%)	Males	Females
Illiterate	-	13 (4.2)
No schooling, literate	5 (2.7)	4 (1.3)
Basic/primary school	22 (12.0)	105 (33.8)
Secondary school	24 (13.1)	29 (9.3)
High school	88 (48.1)	107 (34.4)
University	44 (24.0)	53 (17.0)
	*χ* ^*2*^ *= 40.035; p = 0.000*	
Energy and Nutrients**, mean (SD)	Males	Females
Energy (kcal)	1906.5 (785.2)	1571.4 (696.2)*
Total protein (g)	69.1 (33.2)	52.3 (22.8)*
Protein (% energy)	14.8 (4.7)	13.6 (3.8)*
Total fat (g)	65.4 (31.0)	57.9 (29.1)*
Fat (% energy)	31.0 (8.3)	33.4 (9.9)*
SFA (g)	23.4 (13.8)	19.5 (11.1)*
MUFA (g)	23.0 (10.8)	20.1 (10.3)*
PUFA (g)	14.8 (10.7)	15.02 (11.2)
Dietary cholesterol (mg)	214.4 (147.7)	167.9 (135.0)*
Carbohydrate (g)	252.3 (116.9)	201.0 (97.9)
Carbohydrate (% energy)	52.7 (9.1)	51.3 (9.8)
Fibre (g)	20.3 (10.3)	18.4 (8.9)*
Vitamin A (μg)	890.3 (637.9)	869.2 (857.6)
Vitamin E (mg)	14.8 (13.2)	15.4 (11.9)
Tiamin (mg)	0.97 (0.8)	0.78 (0.4)*
Riboflavin (mg)	1.43 (1.5)	1.07 (0.4)*
Niasin (mg)	12.2 (8.1)	9.4 (5.9)*
Vitamin B_6_ (mg)	1.3 (0.6)	1.2 (0.5)*
Folate (μg)	321.0 (142.1)	281.9 (127.9)*
Vitamin B_12_ (μg)	3.5 (3.4)	2.4 (1.9)*
Vitamin C (mg)	99.7 (89.6)	105.9 (84.1)
Calcium (mg)	603.6 (341.5)	521.3 (268.2)*
Iron (mg)	11.3 (7.6)	9.6 (4.9)*
Zinc (mg)	9.5 (5.5)	7.5 (3.4)*

*p < 0.05, Independent-Sample *T*-Test; **According to the 24-h dietary recall

*BMI* body mass index, *SD* standard deviation, *SFA* saturated fatty acid, *MUFA* monounsaturated fatty acid, *PUFA* polyunsaturated fatty acid

### Comparison of diet quality according to two version of Healthy Eating Indices

The percentage of individuals in the “needs improvement” diet category was 68.0% according to HEI-2005 scores while this ratio were 29.4% in HEI-2010 (κ = 0.302-*fair agreement*; *p* = 0.000; data not shown in the tables). Subjects have good diet quality were found at the ratio of 1.4% in HEI-2005 (data not shown in the tables). There was no individual had good diet quality according to HEI-2010 scores.

The mean total scores and diet quality indices’ components of HEI-2005 and HEI-2010 according to gender, age group, education level and obesity status of individuals were shown in Table 2. The mean scores HEI-2005 and HEI-2010 of individuals were found 56.1 ± 13.9 and 41.5 ± 13.7, respectively (data not shown in the tables). It was determined that females had higher mean total scores than males and differences between groups were significant in both indices (*p* < 0.05) (Table 2).

**Table 2:** The mean total scores and diet quality index component of HEI-2005 and HEI-2010 according to gender, age group, education level and obesity status of individuals

	Gender		Education level (years)		Age group (years)			BMI classification		
	Males	Females	≤8	>8	19-30	31-50	>51	Underweight	Normal	Overweight
	(n:183)	(n:311)	(n:202)	(n:292)	(n:236)	(n:226)	(n:32)	(n:24)	(n:245)	(n:225)
HEI-2005	Mean ± SD	Mean ± SD	Mean ± SD	Mean ± SD	Mean ± SD	Mean ± SD	Mean ± SD	Mean ± SD	Mean ± SD	Mean ± SD
Total fruit	2.2 ± 1.9	2.7 ± 2.0*	2.7 ± 1.9	2.4 ± 2.0	2.4 ± 2.0^a^	2.5 ± 1.9^a,b^	3.2 ± 1.8^b^	2.6 ± 2.3	2.4 ± 2.0	2.6 ± 1.9
Whole fruit	2.9 ± 2.2	3.3 ± 2.1	3.4 ± 2.0	2.9 ± 2.2*	2.9 ± 2.2^a^	3.2 ± 2.1^b^	4.0 ± 1.8^b^	2.9 ± 2.2	2.9 ± 2.2	3.4 ± 2.0
Total vegetables	2.5 ± 1.8	3.1 ± 1.8*	3.0 ± 1.7	2.8 ± 1.8	2.7 ± 1.8	3.0 ± 1.8	3.5 ± 1.8	2.7 ± 1.7	2.7 ± 1.8	3.1 ± 1.8
Dark green and orange vegetables and legumes	2.1 ± 1.9	2.6 ± 2.1*	2.7 ± 2.1	2.2 ± 2.0*	1.9 ± 1.9^a^	2.8 ± 2.0^b^	3.4 ± 2.1^b^	1.7 ± 1.9^a^	1.9 ± 1.9^a^	2.9 ± 2.1^b^
Whole grains	4.9 ± 0.1	4.8 ± 0.6*	4.9 ± 0.6	4.9 ± 0.3	4.9 ± 0.4	4.9 ± 0.5	4.9 ± 0.5	4.9 ± 0.3	4.9 ± 0.3	4.9 ± 0.6
Total grains	0.0 ± 0.0	0.1 ± 0.3	0.0 ± 0.4	0.0 ± 0.0	0.0 ± 0.3	0.0 ± 0.0	0.0 ± 0.0	0.0 ± 0.0	0.0 ± 0.0	0.0 ± 0.3
Milk	2.9 ± 2.5	3.4 ± 2.9	3.2 ± 2.9	3.2 ± 2.7	2.8 ± 2.6^a^	3.6 ± 2.8^b^	4.0 ± 3.1^b^	2.2 ± 2.3	3.1 ± 2.6	3.5 ± 2.9
Meat and beans	6.6 ± 3.7	6.4 ± 3.9	6.3 ± 4.0	6.6 ± 3.7	6.3 ± 3.9	6.7 ± 3.8	5.9 ± 4.1	6.5 ± 3.7	6.2 ± 3.8	6.7 ± 3.9
Oils	6.9 ± 4.6	7.6 ± 4.3	7.5 ± 4.4	7.2 ± 4.5	6.7 ± 4.7^a^	7.8 ± 4.2^b^	8.8 ± 3.4^b^	7.5 ± 4.4^a,b^	6.8 ± 4.7^a^	7.9 ± 4.1^b^
Saturated fats	6.9 ± 2.1	6.8 ± 2.0	6.9 ± 1.9	6.7 ± 2.1	6.5 ± 2.0^a^	7.0 ± 2.0^b^	7.6 ± 1.9^b^	6.3 ± 1.9^a,b^	6.5 ± 2.0^a^	7.2 ± 2.0^b^
Sodium	2.3 ± 3.6	4.1 ± 4.3*	4.1 ± 4.2	2.9 ± 4.0*	3.6 ± 4.2	3.1 ± 4.1	4.0 ± 4.2	4.8 ± 4.4	3.7 ± 4.1	3.0 ± 4.1
Calories from SOFAAS	13.4 ± 8.9	16.6 ± 7.0*	16.1 ± 7.5	14.9 ± 8.2	13.9 ± 8.7^a^	16.6 ± 7.0^b^	18.0 ± 5.9^b^	14.9 ± 8.5	14.7 ± 8.3	16.3 ± 7.4
Total HEI-2005 score	54.6 ± 16.4	62.9 ± 15.0*	58.3 ± 13.6	54.7 ± 14.0*	52.8 ± 14.9^a^	58.5 ± 12.5^b^	64.0 ± 8.7^b^	55.4 ± 9.2^a,b^	53.9 ± 14.7^a^	58.6 ± 13.1^b^
HEI-2010	Mean ± SD	Mean ± SD	Mean ± SD	Mean ± SD	Mean ± SD	Mean ± SD	Mean ± SD	Mean ± SD	Mean ± SD	Mean ± SD
Total fruit	2.2 ± 1.9	2.7 ± 2.0*	2.7 ± 1.9	2.4 ± 2.0	2.4 ± 2.0^a^	2.5 ± 1.9^a,b^	3.2 ± 1.8^b^	2.6 ± 2.3	2.3 ± 2.0	2.6 ± 1.9
Whole fruit	2.9 ± 2.2	3.3 ± 2.1	3.4 ± 2.0	2.9 ± 2.2*	2.9 ± 2.2^a^	3.2 ± 2.1^b^	4.0 ± 1.8^b^	2.9 ± 2.2	2.9 ± 2.2	3.4 ± 2.0
Total vegetables	2.5 ± 1.8	3.1 ± 1.8*	3.0 ± 1.7	2.8 ± 1.8	2.7 ± 1.8	3.0 ± 1.8	3.5 ± 1.8	2.7 ± 1.7	2.7 ± 1.8	3.1 ± 1.8
Greens and beans	2.8 ± 2.1	3.2 ± 2.1*	3.3 ± 2.1	2.9 ± 2.1*	2.7 ± 2.1^a^	3.3 ± 2.0^b^	3.9 ± 1.8^b^	2.7 ± 2.3^a,b^	2.7 ± 2.1^a^	3.4 ± 1.9^b^
Whole grains	0.0 ± 0.0	0.1 ± 0.3	0.0 ± 0.4	0.0 ± 0.0	0.0 ± 0.3	0.0 ± 0.0	0.0 ± 0.0	0.0 ± 0.0	0.0 ± 0.0	0.0 ± 0.3
Dairy	2.6 ± 1.7	2.7 ± 1.8	2.7 ± 1.8	2.6 ± 1.8	2.4 ± 1.7^a^	2.9 ± 1.8^b^	3.2 ± 1.9^b^	1.9 ± 1.6	2.6 ± 1.8	2.8 ± 1.8
Total protein foods	3.6 ± 1.6	3.5 ± 1.8	3.5 ± 1.8	3.6 ± 1.7	3.4 ± 1.8	3.7 ± 1.7	3.5 ± 1.7	3.7 ± 1.5	3.4 ± 1.8	3.7 ± 1.7
Seafood and plant proteins	3.3 ± 2.2	3.7 ± 2.0	3.7 ± 2.0	3.4 ± 2.1	3.2 ± 2.2^a^	3.8 ± 1.9^b^	4.3 ± 1.6^b^	3.07 ± 2.3^a,b^	3.3 ± 2.2^a^	3.9 ± 1.9^b^
Fatty acids	0.1 ± 0.5	0.2 ± 1.2*	0.2 ± 1.2	0.2 ± 0.9	0.2 ± 1.1	0.2 ± 0.9	0.0 ± 0.1	0.0 ± 0.0	0.2 ± 0.9	0.2 ± 1.1
Refined grains	0.9 ± 1.7	1.6 ± 2.1*	1.2 ± 1.9	1.4 ± 2.0	1.4 ± 2.0	1.3 ± 1.9	1.4 ± 1.9	2.3 ± 2.4^a^	1.3 ± 1.9^b^	1.3 ± 1.9^b^
Sodium	2.3 ± 3.6	4.1 ± 4.3*	4.1 ± 4.2	2.9 ± 4.0*	3.6 ± 4.2	3.1 ± 4.1	4.0 ± 4.2	4.8 ± 4.4	3.7 ± 4.1	3.0 ± 4.1
Empty calories	13.3 ± 8.9	16.5 ± 7.0*	15.9 ± 7.5	14.8 ± 8.2	13.8 ± 8.7^a^	16.5 ± 7.0^b^	17.9 ± 5.9^b^	14.7 ± 8.4	14.5 ± 8.3	16.2 ± 7.4
Total HEI-2010 score	37.4 ± 15.3	46.1 ± 13.9*	43.9 ± 13.2	40.0 ± 13.9*	38.7 ± 14.6^a^	43.6 ± 12.4^b^	48.9 ± 10.3^b^	41.5 ± 9.5^a,b^	39.7 ± 14.4^a^	43.7 ± 13.1^b^

*p < 0.05 for the data classified into two categories, ANOVA

^a,b^Different characters *p* < 0.05, same characters *p* > 0.05 for the data classified into three categories

According to both versions of diet quality indices, females had significantly higher scores than that of males in some components including consumption of total fruits and vegetables, dark green and orange vegetables and legumes (HEI-2005), greens and beans (HEI-2010). Scores obtained from “sodium” and “calories from SoFAAS” were lower in males than that of females according to HEI 2005 and likewise, the mean scores of refined grains, sodium, fatty acids and empty calories was lower in males than that of females according to HEI-2010 (*p* < 0.05). Mean scores of whole fruits, whole grains, milk, meat and beans, saturated fats and oils in the HEI-2005 and mean scores of whole grains, dairy, whole fruit, total protein foods, seafood and plant protein in the HEI-2010 were similar in male and female. There were no significant differences between groups (*p* > 0.05).

It was found that individuals with lower education level had higher scores from whole fruit, dark green and orange vegetables, legumes and sodium intake in both versions (*p* < 0.05).

The youngest age group (19–30 years) had lowest scores of both for HEI-2005 (52.8 ± 14.9) and for HEI-2010 (38.7 ± 14.6) and the differences between other age groups were significant (*p* < 0.05). The mean scores from whole fruits, dark green and orange vegetables and legumes, milk, saturated fats, oils and energy from SoFAAS in the HEI-2005 was found lowest in 19–30 ages group and differences between other age groups were significant (*p* < 0.05). And also the mean scores from whole fruits, greens and beans, seafood and vegetable protein foods, dairy with empty calories in the HEI-2010 was found lowest in 19–30 ages group and differences between other age groups were significant (*p* < 0.05).

Mean total scores of both versions of HEI were higher among overweight individuals than normal individuals in the diet quality indices. While there was not any significant difference between underweight and overweight individuals in total scores of HEI-2005 and HEI-2010 (*p* > 0.05) but difference between normal and overweight individuals was significant (*p* < 0.05).

When diet quality of individuals was assessed according to gender, age groups, education level and BMI classification it was found that individuals needed improvement their diet quality according to HEI-2005 scores and their diet quality was poor according to HEI-2010 scores in all groups of age, education levels and BMI.

### Diet quality of individuals according to baseline characteristics

Baseline characteristic of the participants by classification of HEI scores was presented in Table 3. Females who have good diet quality were more than males in HEI-2005 (*χ* ^*2*^ = 18.990; *p* = 0.000). The percentages of males in the “needs improvement” and “poor” diet categories were 57.4% and 42.1%, respectively. The percentage of females who had poor diet quality were lower (64.3%) than males according to HEI-2010 (*χ* ^2^ = 16.269; *p* = 0.000) (Table 3). The ratio of participants have poor diet quality was 34.6% in high education level (>8 years) while this ratio was 24.8% in low education level according to HEI-2005. But the difference was not significant (*p* > 0.05). Percentage of participants in the “needs improvement” diet category was higher than the ratios of other diet categories in all age groups according to HEI-2005. According to HEI-2010, in only “≥51 years” age group, diet quality of the most individuals was classified as “need improvement”. The percentages of participants who have poor diet quality were higher significantly in the other age group than that of “≥51 years” age group. Overweight participants who have poor diet quality were less (22.2%) than underweight (38.0%) and normal subjects (33.3%) (*χ* ^2^ = 15.947; *p* = 0.003) according to HEI-2005. There was no significant difference between normal and underweight individuals (*p* > 0.05). Similarly according to HEI-2010 overweight subjects in “poor” diet quality category were less (64.0%) than the others (*χ* ^2^ = 10.426; *p* = 0.005). Also, most of the overweight subjects’ diet qualities were evaluated to need improvement according to HEI-2005 while the most overweight individuals were evaluated to have poor diet quality in HEI-2010. Components and scoring standards of HEI-2005 and HEI-2010 were presented in Table 4. There are some changes in the evaluation standards of the HEI-2005 when developing the HEI-2010. For example, ≥12 g oil per 1000 kcal is required for a maximum score of 10 according to HEI-2005, while in HEI-2010, for a maximum score of 10, the standard is presented that sum of polyunsaturated fatty acids (PUFAs) and monounsaturated fatty acids (MUFAs) to saturated fatty acids (SFAs) ratio of greater than two and a half [(PUFAs + MUFAs)/SFAs >2.5]. This change encourages healthy choices of protein and fat. Also, HEI-2010 gives more attention to food quality than HEI-2005. HEI-2010 includes only whole grains instead of total grains contrary to HEI-2005.

**Table 3:** Evaluation of diet quality of individuals according to healthy eating indices by gender, education level, age group and BMI classification, *n* (%)

	Gender		Education Level (years)		Age group (years)			BMI classification		
	Males	Females	≤8	>8	19-30	31-50	≥51	Underweight	Normal	Overweight
HEI-2005										
Poor	77 (42.1)	74 (23.8)	50 (24.8)	101 (34.6)	93 (39.4)^a^	54 (23.9)^b^	4 (12.5)^b^	8 (33.3)^a, b^	93 (38.0)^a^	50 (22.2)^b^
Needs improvement	105 (57.4)	231 (74.3)	149 (73.8)	187 (64.0)	138 (58.5)^a^	170 (75.2)^b^	28 (87.5)^b^	16 (66.7)^a, b^	147 (60.0)^a^	173 (76.9)^b^
Good	1 (0.5)	6 (1.9)	3 (1.5)	4 (1.4)	5 (2.1)^a^	2 (0.9)^b^	-	-	5 (2.0)^a^	2 (0.9)^b^
	*χ* ^*2*^ *= 18.990; p = 0.000*		*χ* ^*2*^ *= 5.450; p = 0.066*		*χ* ^*2*^ *= 21.138; p = 0.000*			*χ* ^*2*^ *= 15.947; p = 0.003*		
HEI-2010										
Poor	149 (81.4)	200 (64.3)	130 (64.4)	219 (75.0)	184 (78.0)^a^	150 (66.4)^b^	15 (46.9)^c^	21 (87.5)^a^	184 (75.1)^a^	144 (64.0)^b^
Needs improvement	34 (18.6)	111 (35.7)	72 (35.6)	73 (25.0)	52 (22.0)^a^	76 (33.6)^b^	17 (53.1)^c^	3 (12.5)^a^	61 (24.9)^a^	81 (36.0)^b^
Good	-	-	-	-	-	-	-	-	-	-
	*χ* ^*2*^ *= 16.269; p = 0.000*		*χ* ^*2*^ *= 6.523; p = 0.011*		*χ* ^*2*^ *= 16.809; p = 0.000*			*χ* ^*2*^ *= 10.426; p = 0.005*		

^a,b,c^Different characters *p* < 0.05, same characters *p* > 0.05 for the data classified into three categories

**Table 4:**
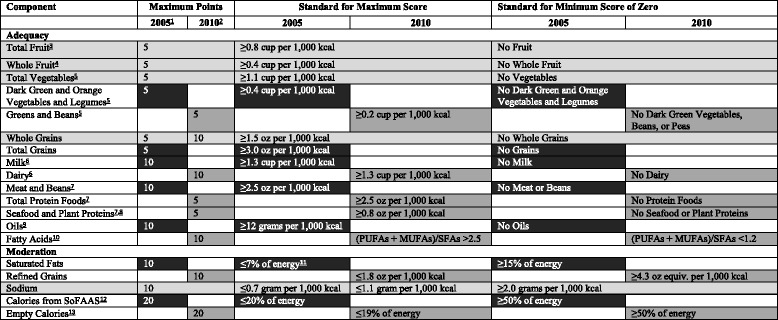
Comparison of components and scoring standards in the HEI-2005 and HEI-2010 [10]

Light gray rows indicate components found in both the HEI–2005 and HEI–2010; Black rows indicate components found only in the HEI–2005; Dark gray rows indicate components found only in the HEI–2010

^a^Intakes between the minimum and maximum standards are scored proportionately except for Saturated Fat and Sodium

^b^Intakes between the minimum and maximum standards are scored proportionately

^c^Includes 100% fruit juice

^d^Includes all forms except juice

^e^Includes any beans and peas (called Legumes in HEI–2005) not counted as Total Protein Foods (called Meat and Beans in HEI–2005)

^f^Includes all milk products, such as fluid milk, yogurt, and cheese, and fortified soy beverages

^g^Beans and peas are included here (and not with vegetables) when the Total Protein Foods (called Meat and Beans in HEI–2005) standard is otherwise not met

^h^Includes seafood, nuts, seeds, soy products (other than beverages) as well as beans and peas counted as Total Protein Foods

^i^Includes non-hydrogenated vegetable oils and oils in fish, nuts, and seeds

^j^Ratio of poly- and monounsaturated fatty acids to saturated fatty acids

^k^Saturated Fat and Sodium get a score of 8 for the intake levels that reflect the 2005 Dietary Guidelines, <10% of calories from saturated fat and 1.1 g of sodium/1,000 kcal, respectively. Intakes between the standards for scores of 0 and 8 and between 8 and 10 are scored proportionately

^l^Calories from solid fats, alcoholic beverages, and added sugars

^m^Calories from solid fats, alcoholic beverages, and added sugars; threshold for counting alcohol is >13 g/1,000 kcal

The correlation of age and BMI with HEI-2005 and HEI-2010 scores of individuals was found to be significantly correlated. There was a positive correlation between both version of HEI with age and BMI values. Besides, HEI-2005 and HEI-2010 scores correlated positively with each other (r = 0.893; *p* = 0.000; data not shown in the tables).

## Discussion

Diet quality of participants was low according to both versions of healthy eating indices and male had lower score than that of female. When comparing HEI-2010 with HEI-2005, males had higher score in HEI-2005 (*p* < 0.05). In a study performing through HEI-2010 and HEI-2005, diet quality was found similar to our study and male had poor diet quality [32]. Similarly, Drewnowski et al. (2016) demonstrated that women had higher quality diets than men according to HEI-2005 (*p* < 0.0001) and similar results were observed for HEI-2010 scores [33]. But another study evaluated diet quality by HEI-2005 there was no significant difference between genders [34]. In another study evaluating the diet quality it was emphasized that individuals with healthy eating habits had higher diet quality index score and lower obesity risk [35]. There are many studies about evaluated relationship between diet quality and BMI [36–39]. It was determined that diet quality index score was negatively correlated with BMI at some studies which are compared BMI and diet quality [35, 40–42]. But in our study, diet quality score of overweight individuals was higher. Similarly, in a prospective cohort study included 50,434 African-Americans, 24,054 white individuals, and 3,084 individuals of other racial/ethnic groups, it has been found that HEI score was positively associated with BMI. Also, according to the same study HEI-2005 has been strongly correlated with HEI-2010 score (r_*p*_ = 0.91) [43]. This association was found in the present study with Pearson correlation coefficient of 0.893. According to Kappa statistical results, the agreement between HEI-2005 and HEI-2010 was statistically significant (κ = 0.302-*fair agreement*).

Hiza et al. (2013) found that there are growing healthy eating consciousness and interest to be protected chronic disease with increasing age [44]. In the present study, it was stated that individuals older than 51 years have more total scores at both versions of healthy eating indices and in another study it was determined that diet quality correlates their ages in this age [32]. Thus, older ages have better diet quality than younger ages [34] similar to our results. Monfort-Pires et al. demonstrated that the mean HEI-2005 score of individuals aged between 18 and 62 years was 65.0 ± 10.8 [45]. This result was higher than that of our study (56.1 ± 13.9) but according to mean score it was emphasized that they have to improve their diet quality similar our study. In this research, subjects have good diet quality were found the ratio of 1.4% in HEI-2005 while there was no individual had good diet quality according to HEI-2010 scores. Similarly, HEI-2010 scores of US population aged 2 and older have been found to be low (49.9 points) [32] based on The National Health and Nutrition Examination Survey (NHANES) data. According to US food supply data, it was demonstrated that the HEI-2010 score ranges from 48 points in 1970 to 55 points in 2010 [46]. In Turkey, there was no study assessed the changes in diet quality from year to year using HEI-2010. So, this study provides an important contribution to the literature.

Personal development at cultural, economic and education field of individuals is inversely associated with diet quality at many studies. The reason of this has been expressed as increased frequency of ready and unhealthy foods with eating out of home. And because of this, consumption of sugar and sodium by individuals with high educational level has been increased [47–49]. Similarly in our study, individuals who have higher education level had lower diet quality. But this result was inconsistent with other studies [33, 34]. The other studies asserted that with the increased education level and awareness of healthy nutrition, diet quality was increased [44].

In present study, it was determined that according to HEI-2005 many individuals (57.5% in males and 74.3% in females) had to improve their diet quality and in many studies were found results similar to our study [50, 51]. When we investigate the components used in the determination of diet quality, it was shown that lower scores obtained from vegetables, fruit and dairy components and these results are very similar in a study conducted in adults [52]. Low intake of vegetables, fruits and dairy adversely affects diet quality and low diet quality often occurs as a result of highly oil consumption [40]. Scores obtained from saturated fats and oils were higher than the other components in HEI-2005 but fatty acids was found to be contributed to total score less than the other components in HEI-2010. This situation arises because of content of the total oils consumed by individuals. Scores of total grain which is subcomponent of HEI-2005 were higher than that of whole grain and there was a significant difference between genders. Total grain score was not present and scores of whole grains was low in HEI-2010. Score obtained from whole grain was very low in another study which was evaluating diet quality similar to our study [30]. Consumption of recommended amounts whole grain is effective at weight control and prevention of chronic diseases. Increasing consumption of whole grain which is subcomponent of the both of indices will contribute to diet quality and improvement of food intake [53]. Also, diet quality is inversely associated with the consumption of foods and drinks, the sources of the “empty calories” [27]. In this research, contribution of “calories from SoFAAS” which is component of HEI-2005 and of “empty calories” which is component of HEI-2010 to total indices scores was greater than other component. Thus, it was found that consumption of foods and drinks containing empty calories obtained from saturated fat, alcohol and added sugar was low in this study.

The present study includes a number of limitations. The major limitation of this cross sectional study include that socioeconomic status didn’t examine that affect the diet quality. But it is difficult to evaluate this variable in Turkey because most of individuals don’t want to give information about their income. This study includes only people who are living in central Anatolia and does not reflect the whole of Turkey. Therefore, studies are needed that are homogeneous, wide and could represent all society.

In conclusion, studies that evaluate the diet quality of individuals using HEI-2005 and HEI-2010 are limited in Turkey. In accordance with the results of this study, diet qualities of individuals are affected by age, gender, education and BMI. Although the mean score of the indices were found to be almost close and they were strongly correlated with each other, HEI-2010 provided more precise results. Also, many individuals’ diets in “needs improvement” group according to HEI-2005 were assessed as “poor” in HEI-2010. At this regard, it should be noted that various indices using for diet quality assessment can lead to differences. Indices provide most precise results and current should be preferred. Also, it may be more useful to support these results with using other dietary assessment methods such as nutrient adequacy ratio, mean adequacy ratio and/or other indices in addition to the HEI.
